# Potential effects of shift work on skin autoimmune diseases

**DOI:** 10.3389/fimmu.2022.1000951

**Published:** 2023-02-14

**Authors:** Sarah Stenger, Hanna Grasshoff, Jennifer Elisabeth Hundt, Tanja Lange

**Affiliations:** ^1^Lübeck Institute of Experimental Dermatology, University of Lübeck, Lübeck, Germany; ^2^Department of Rheumatology and Clinical Immunology, University of Lübeck, Lübeck, Germany; ^3^Center for Research on Inflammation of the Skin, University of Lübeck, Lübeck, Germany; ^4^Center of Brain, Behavior and Metabolism (CBBM), University of Lübeck, Lübeck, Germany

**Keywords:** shift work, skin, sleep, circadian, autoimmune, cortisol, melatonin, inflammation

## Abstract

Shift work is associated with systemic chronic inflammation, impaired host and tumor defense and dysregulated immune responses to harmless antigens such as allergens or auto-antigens. Thus, shift workers are at higher risk to develop a systemic autoimmune disease and circadian disruption with sleep impairment seem to be the key underlying mechanisms. Presumably, disturbances of the sleep-wake cycle also drive skin-specific autoimmune diseases, but epidemiological and experimental evidence so far is scarce. This review summarizes the effects of shift work, circadian misalignment, poor sleep, and the effect of potential hormonal mediators such as stress mediators or melatonin on skin barrier functions and on innate and adaptive skin immunity. Human studies as well as animal models were considered. We will also address advantages and potential pitfalls in animal models of shift work, and possible confounders that could drive skin autoimmune diseases in shift workers such as adverse lifestyle habits and psychosocial influences. Finally, we will outline feasible countermeasures that may reduce the risk of systemic and skin autoimmunity in shift workers, as well as treatment options and highlight outstanding questions that should be addressed in future studies.

## 1 Introduction

Our 24/7 society leads to an increase in shift work with work schedules that fall outside the standard work hours from 7 AM to 6 PM. Shift work includes early morning, evening, or night shifts, as well as fixed or rotating shifts. About 15-25% of the world-wide population is working in shifts (1), in particular health care workers. In this latter population epidemiological studies provide alarming data on shift workers showing increased rates of longstanding illnesses (2). In addition, the Nurses’ Health Study demonstrated enhanced mortality due to cardiovascular diseases or lung cancer (3). Shift workers are forced to be active and to sleep at time periods that are out of sync with their endogenous time-keeping system. This internal clock, the circadian system, is comprised of clock genes in many, if not all cells of the body. The molecular machinery of cellular clocks involves -among others- the core clock genes Brain and Muscle ARNT-Like1 (in humans *BMAL1*, in mice *Bmal1*) and Circadian Locomotor Output Cycles Kaput (*CLOCK, Clock*), which form dimers upon translation and initialize the transcription of Period (*PER, Per*) genes. These form a dimer with Cryptochrome (*CRY, Cry*) genes. This in turn inhibits the transcription of *BMAL1*. Moreover, the BMAL1-CLOCK-dimer is binding together with the dimer of *NFIL3* and *DBP* to the E box of *REVERB*/*Reverb* genes, initiating their transcription. In turn, *REVERB*/*Reverb* genes inhibit the transcription of *NFIL3*/*Nfil3*, forming another feedback-loop (4). With the interaction of other genes and proteins, a network of interconnected loops is formed, taking approximately 24 hours (h) to be executed. The master clock in the hypothalamic suprachiasmatic nuclei (SCN) is synchronized to external time cues like 24 h light-dark changes, a process that is called entrainment (4). In turn, the SCN entrain peripheral clocks by systemic signals such as core body temperature, mediators of the stress systems (such as the sympathetic nervous system (SNS) and the hypothalamus-pituitary-adrenal (HPA)-axis, as well as melatonin, the pineal hormone of darkness (5). The circadian system controls virtually all body functions like sleep and wakefulness, behavioral changes in physical activity and food intake, thermoregulation, cell proliferation and metabolism, the cardiovascular, the endo-crine, the digestive, the reproductive and the immune system (6–16). Apart from light, several non-photic external time cues have been described that were summarized in a review by Mistlberger and Skene (17). Exemplarily, food is a non-photic external time cue that can feedback to the SCN to further entrain 24 h rhythms

When shift workers experience a mismatch of their internal clock with environmental cues and obligations this circadian misalignment can result in circadian disruption of body functions from the molecular to the behavioral level. Potential outcomes are poor sleep, chronic stress (18), burn out syndrome (19), social isolation (20) and adverse lifestyle habits like physical inactivity (18), unhealthy diet (21, 22), or substance abuse (23). All these processes can trigger systemic chronic inflammation (SCI) (24, 25) and a dysregulation of innate and adaptive immune responses. That alone or in combination may foster infectious, cardiovascular, metabolic and cancer diseases in shift workers (22, 26, 27). In addition, the STRESSJEM Study, which was conducted in France, analyzed mortality and cause-specific mortality due to night and shift work. Niedhammer et al. described a sex-specific association between night and shift work and cerebrovascular diseases, ischemic heart diseases, respiratory cancers and breast cancer as causes of mortality (27). Thus, a life against the internal clock can impair host defense against pathogens (28, 29) and tumors (30) and also seems to promote unwanted immune responses to harmless antigens, like allergens (31) or auto-antigens (32). While shift workers are at higher risk to develop systemic autoimmune diseases as shown for rheumatoid arthritis (RA) (33), data on associated skin manifestations or skin-specific autoimmune diseases is only limited. In the following, we will outline that shift work likely impairs skin physiology and immunity and thus could promote skin autoimmune diseases, a causality that, however, needs to be experimentally clarified in future studies.

In detail, we will first describe 24 h in the life of a physician during a night shift in a narrative in section 2 and outline (**Table 1**) experimental approaches to delineate the effects of shift work on the skin and the immune system in section 3. In section 4, we will then summarize epidemiological and experimental evidence in humans and animals indicating that shift work could promote skin autoimmune diseases, before we give more detailed reports on the cellular effects of shift work on skin physiology, skin innate immunity and skin adaptive immunity in section 5. In section 6, we will discuss candidate neuroendocrine mediators linking shift work with skin autoimmune diseases and in section 7 we will highlight potential countermeasures and therapeutic approaches to prevent, ameliorate or treat skin autoimmune diseases in shift workers. In the last part of our review, in section 8, we will summarize the outlined findings and give an outlook on outstanding questions that should be addressed in future experiments

## 2 24 hours in the life of Dr. S.W.

In a narrative describing 24 h in the life of an intensive care unit physician who we called Dr. S.W. on night shift, we would like to outline shift work-induced changes in behavior, neuroendocrine mediators, thermoregulation, skin physiology and immune functions in comparison to regular 24 h rhythms. We hypothesize that fine-tuned physiological rhythms in neuroendocrine-immune interactions foster skin barrier functions and that disturbances thereof could promote patho-physiological processes of skin autoimmune diseases. We chose a tabular form with 4 h intervals (**Table 1**) (34–55).

**Table 1:** 24 hours in the life of Dr. S.W. on night shift.

**1 PM**	When Dr. S.W. went to bed after her second night shift it took a while until she fell asleep. Her thermoregulation is out of phase, and she consumed several cups of coffee. Both aspects likely interfere with sleep initiation, maintenance and deep sleep (34). Normally, she would have lunch now, her stress systems would be active, and the bright light of the midday sun would suppress her melatonin levels (35). In bed however, the light that breaks through the window sealings and the noise from outside fragment and shallow her sleep.
**5 PM**	Dr. S.W. woke up but does not feel well rested or refreshed. Presumably, she lacked slow wave sleep (SWS), the deepest form of sleep that normally helps to flush away metabolites from the brain parenchyma (36) and to clear sleep regulatory substances like adenosine, tumor necrosis factor (TNF) and interleukin-1 (IL-1) that induce fatigue and sleepiness (37). When she looks into the mirror, she notices that her skin is pale and that she has an unhealthy appearance. These are findings that also emerge after experimental sleep deprivation (38). Sleep supports anti-oxidative and regenerative processes and a lack thereof impairs skin integrity (39). Moreover, sleep loss is associated with systemic chronic inflammation (SCI) that is a likely mechanism of fatigue, sleepiness, bad mood, cognitive impairments and other feelings and symptoms of sickness (40). It is getting dark already and Dr. S.W.’s breakfast consists of three cups of coffee and, as she failed to buy groceries, a chocolate bar. She comforts herself that caffeine not only antagonizes the sleep-inducing substance adenosine (41) but may also counteract SCI that evolved due to sleep loss (42). Maybe she should take some vitamin D as well to fight against the inflammatory processes (43).
**9 PM**	Dr. S.W. heads to the clinic for her third night shift and takes the car instead of her bicycle. In the doctor’s room she switches on all the lights and drinks a cup of coffee to become alert (34). Normally, her melatonin would rise, and her stress systems would calm down at this time of the day (35). These changes would induce an increase in skin temperature, a decrease in core body temperature and in this way her body would get prepared for the sleep period (34, 44, 45).
**1 AM**	In the patient rooms and the ward corridor the light is dimmed, and the volume of the alarm sounds were turned down. The intensive care unit (ICU) staff generally agrees that the patients should sleep at night to recover (46). On a regular wake-sleep cycle also Dr. S.W. would be in deep SWS now. Her immune system would be boosted by increases in growth hormone, prolactin and aldosterone and very low cortisol and catecholamine levels (35, 47, 48). These hormonal changes presumably also support anabolic processes like cell proliferation and cell growth, as well as anti-oxidative and regenerative processes (49, 50). However, Dr. S.W.’s hormone secretion is disturbed.
**5 AM**	Dr. S.W. is freezing. Normally, her core body temperature would be at minimum now and her internal clock would increase the propensity of rapid eye movement (REM) sleep. Neurotransmitters of the sympathetic nervous system (SNS) such as the catecholamines epinephrine and norepinephrine now would reach nadir levels (51). For the staff of the ICU this night, the opposite holds true. Dr. S.W. hears a red alarm. A patient has a cardiac arrest, and she starts resuscitation. After stabilizing the patient, the ICU team sits together, they drink coffee and eat potato chips. They agree that night shifts favor unhealthy diets (18, 52), substance abuse (23), social isolation (20) and TV time and that these adverse lifestyle habits may increase mortality (53).
**9 AM**	Dr. S.W.’s internal clock activated her stress systems, leading to reduced feelings of fatigue and sleepiness. She drives back home and reflects her life. She loves being at the ICU. It is a meaningful work and there is no doubt about the necessity of 24/7 shifts in contrast, to e.g., night shifts in the supermarket. However, increasing economic pressure in health care leads to displacements of routine procedures into the evening and night hours with adverse consequences for health care workers, their performance and their stress levels and for patient outcomes (54, 55). When she gets out of the car, she wonders whether seeking a specialization in dermatology or rheumatology would be a healthier career option.

## 3 Experimental approaches to delineate the effects of shift work on the skin and the immune system

In this chapter we aim to describe the different types of studies in humans as well as in animals, which can be used to assess the effects of shift work on the skin and the immune system.

### 3.1 Epidemiological and in-laboratory studies in humans

The circadian system controls skin physiology (56) as well as leukocyte ontogeny, differentiation, traffic, and function and thus various aspects of innate and adaptive immunity (57, 58).

Skin physiology in humans can be assessed *in vivo* non-invasively by inspection, by photo documentation, by imaging techniques like optical coherence tomography (59), as well as by measurements of skin temperature, skin pH, skin conductance, or transepidermal water loss (60). Skin physiology can also be measured invasively by harvesting suction blister fluid, by skin biopsies, or by injecting substances that induce an observable skin reaction.

Delayed type hypersensitivity (DTH) describes the cell mediated allergic immune reaction to a certain substance. This takes several days to develop as it involves antigen presenting cells as well as T helper 1 cells (Th1) and T helper 17 (Th17) cells. These recognize the antigen and release cytokines, attracting cytotoxic T cells, which kill the target cells (61, 62). A DTH-reaction also occurs in several autoimmune diseases such as RA where collagen is attacked as well as thyroiditis with the thyroglobulin antigen as a target (63, 64).

Immune parameters in humans are mainly assessed in blood (e.g., numbers of certain leukocyte subsets, levels of cytokines) and immune functions can be tested *in vitro* by using leukocyte cell lines, *ex vivo* by culturing freshly sampled blood leukocytes or *in vivo* by administering immunomodulatory substances and measuring the emerging immune response (e.g., antibody response to vaccination). The assessment of innate and adaptive immunity of the skin requires the invasive methods described above that allow the measurement of immune cells or mediators in fluids or tissues (e.g., histology, immunohistochemistry, immunofluorescence, fluorescent activated cell sorting, enzyme linked immunosorbant assay (ELISA), Western blotting).

The circadian system is closely linked to sleep that itself has manifold effects on skin health and skin ageing (65) and the immune system (47). 24 h rhythms of human behavior including sleep-wake behavior are monitored by questionnaires or wearables like actigraphy watches (66, 67). The latter can also track ambient light and temperature, heart rate, skin temperature and skin conductance. The gold standard to measure sleep with its different stages from light sleep to deep sleep (slow wave sleep (SWS)) and rapid eye movement (REM) sleep is polysomnography, encompassing electroencephalography for brain activity, electrooculography for eye movements and electromyography for muscle activity. It can be recorded with ambulatory devices in the home setting or in the sleep laboratory, where it can be combined with videotaping, monitoring of core body temperature and repeated blood sampling. The two-process model of sleep regulation describes the control of the onset, duration and quality of sleep, as well as increases in alertness and performance during wakefulness. The homeostatic “process S” involves sleep regulatory substances such as adenosine, tumor necrosis factor (TNF) and interleukin (IL)-1. This is combined with the circadian “process C”, which regulates wakefulness by wake-promoting neurotransmitters such as catecholamines (68, 69). Interestingly, the likelihood to fall asleep is highest, when temperature of the distal skin regions (e.g., fingers and toes) is rising in the evening (44). Further, sleep can be deepened by passive warming of the skin (70). Apart from the circadian regulation of sleep by process C, sleep can feedback to the circadian system and impact body rhythms on the level of the SCN (71–73) and the periphery (74–78). Consequently, 24 h rhythms in skin physiology or a given immune parameter, could stem from the effects of the circadian system, from sleep, or both. Moreover, experimental manipulation of the circadian system likely changes sleep and *vice versa*, experimental manipulation of sleep can impact 24 h rhythms of skin and immune parameters.

Along this line, it has been shown in cross-sectional and longitudinal epidemiological studies that shift work induces both circadian disruption and sleep disturbances (79, 80). About 20-30% of shift workers even suffer from shift work disorder, a primary circadian rhythm sleep disorder with debilitating sleep disturbances and/or excessive sleepiness (81). Notably, also other primary sleep disorders (e.g., obstructive sleep apnea) and secondary sleep complaints due to comorbidities (e.g., depression) or medication (e.g., steroids) should be ruled out when studying shift work-autoimmune relationships (82, 83). To mimic shift work, circadian disruption can be induced in healthy volunteers experimentally by changes of the light-dark cycle, mistimed food intake, or mistimed sleep by delaying, depriving or fragmenting sleep (84, 85). Comparable experimental procedures in animals to directly manipulate the circadian system or sleep on the cellular level will be summarized in the next section.

### 3.2 Animal models to study interactions between the circadian system, sleep, and the immune system

**Figure 1:**
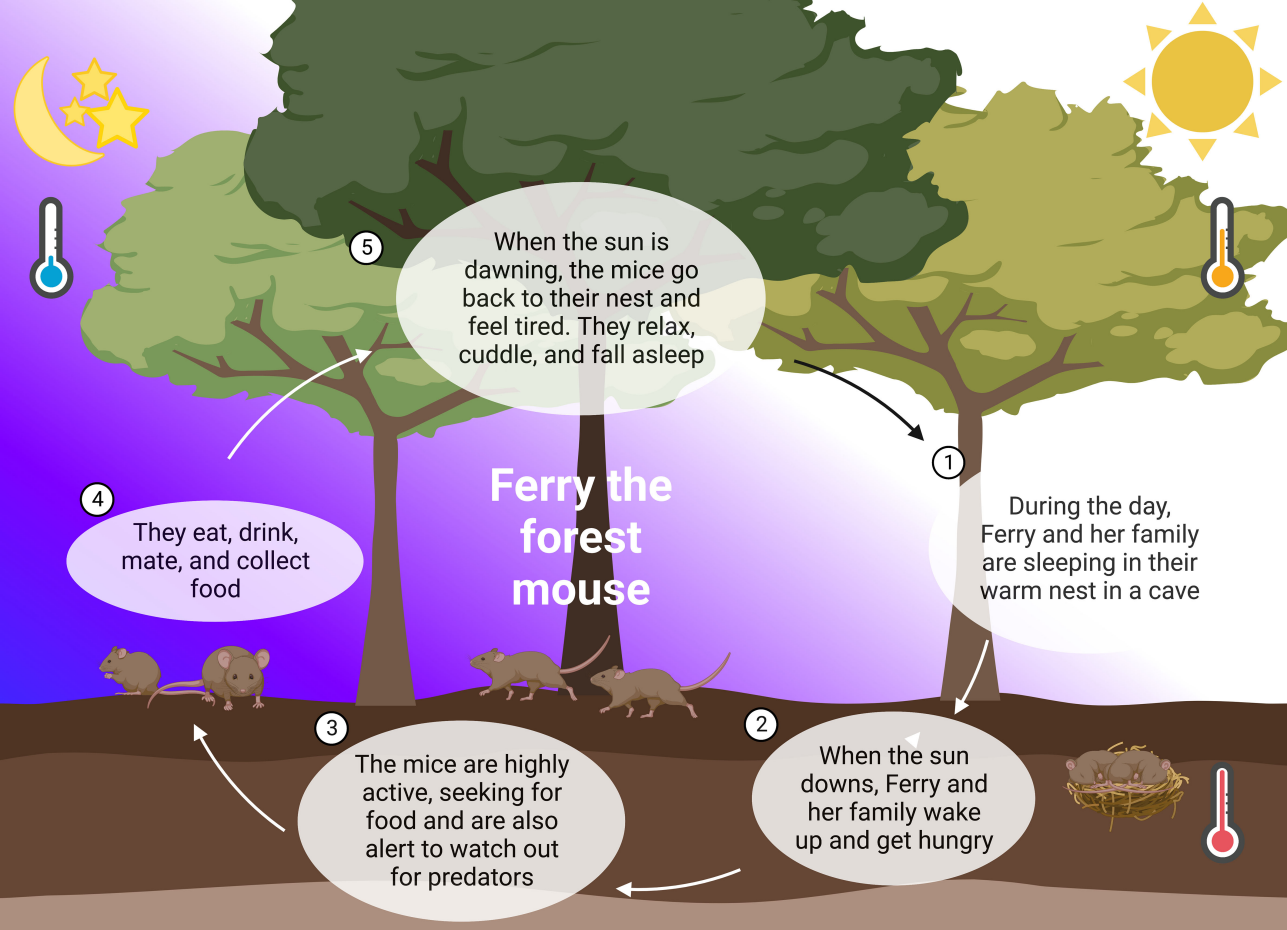
24 hours in the life of Ferry the forest mouse. In nature, mice follow their natural rhythm guided by abiotic *zeitgebers* such as light and temperature. They sleep during the day, cuddling in their warm nest in the soil (**1**). When the sun is downing, they wake up and leave the nest (**2**). The mice use the night and their highest alertness to seek for food and watch out for predators (**3**). A lot of running, climbing, and collecting of food during the night (**4**), releases them happily tired into the day and their sleep (**5**).

**Figure 2:**
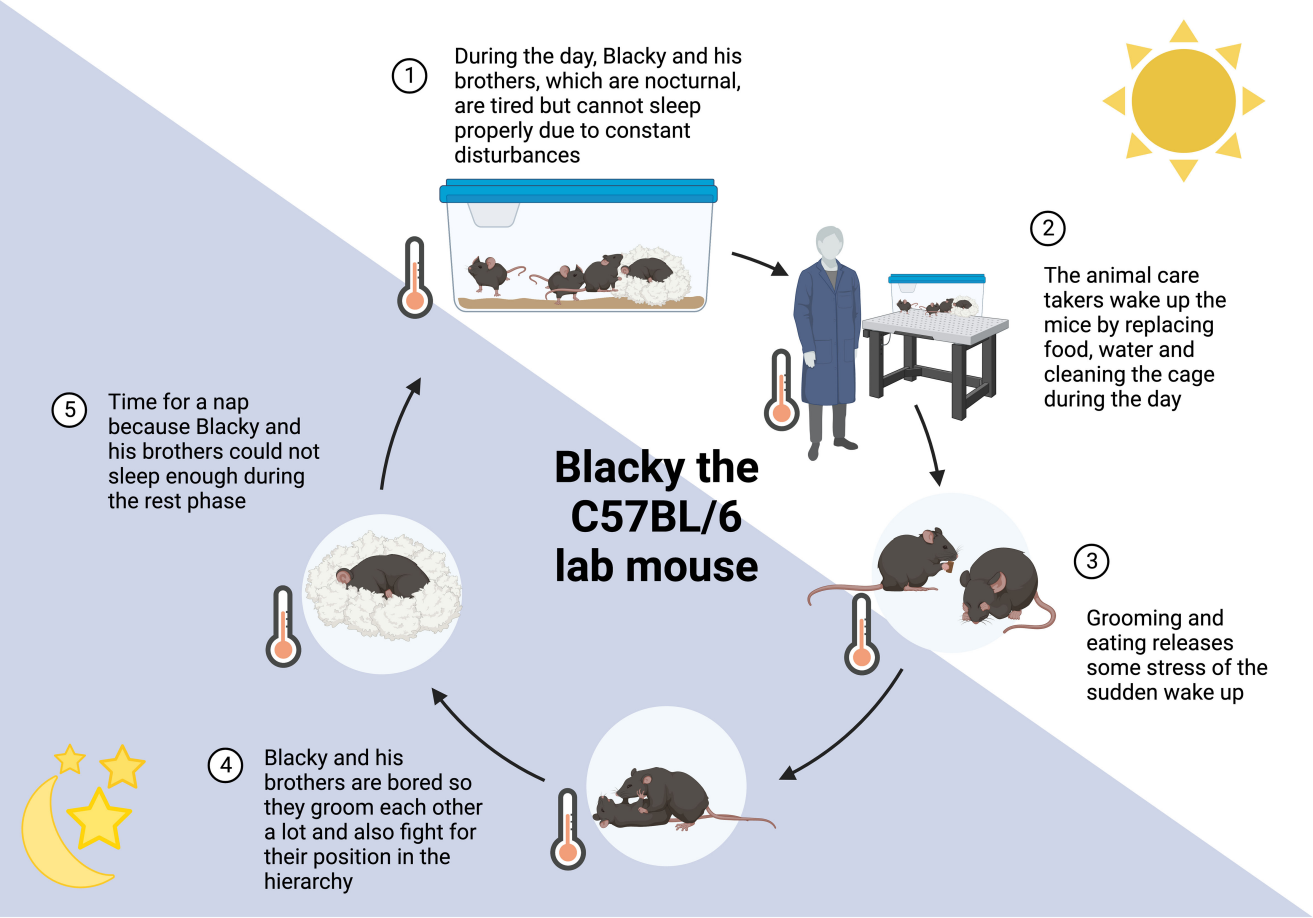
24 hours in the life of Blacky the lab mouse. Mice are nocturnal animals, being active during the night, which is opposite to humans. This leads to various interruptions of their rest period (**1**, **2**), accompanied by stress and changes in behavior. Grooming and eating alleviates some stress caused by sleep deprivation (**3**). The cage-environment is highly restricted in space, movement, and explorative options, resulting in coping behavior (**4**). Moreover, mice might catch up some sleep that was missed in the rest period (**5**). More information can be found in the **Box 1**.

Most animal experiments in biomedical research are performed in mice that are active at night and sleep during the day (86). Wild mice are orientating on *zeitgebers* such as the light/dark cycle, ambient temperature and seasonal dynamics (87). During the day, the mice are asleep and when the sun is downing and the temperature is lowering, this is the signal to get awake and be active (**Figure 1**). These wild mice are exposed to a completely different life and stressors (87) than their counterparts in the laboratories. They have many more options to explore, more space to run around and also larger territories than in a standard cage. The social groups form dynamically and are not gender specific (88). Wild mice are rarely disturbed during the rest phase; however, they have to cope with the stressors of predators, pathogens and limited access to food (87, 89, 90). Unfortunately, these natural conditions cannot be mimicked in the laboratory setting (91) (**Figure 2** and **Box 1**) (92–98).

**BOX 1 Blacky, the lab mouse (Figure 2).**

Blacky, a C57BL/6 mouse, is living with his brothers in a ventilated cage with 501 cm^2^. Their home is comfortable with a fluffy nest. During the day, when the light is turned on, all brothers want to sleep and cuddle in their nest to stay warm and get some rest (**1**). It is not as cozy as it sounds because there is constantly noise from humans around them and the ambient temperature is below the brothers’ thermoneutral zone of 30°C. The temperature of 22°C is constant for 24 hours, which makes it even more difficult to decide whether it is time to be awake or asleep, it is just always cold. From time to time, their entire home is picked up while they are asleep and put somewhere else or the nest is exchanged for a new nest. Somedays even all brothers are woken up and placed in a new home (**2**). These days are truly horrible and to compensate the stress, the brothers groom each other, even plucking off the whiskers or entire patches of fur. Some of the brothers also get hungry or feel the urge to chew to get rid of the stress (**3**) (92, 93). So, a little meal in between is very common and especially Black-Jack, the biggest brother who leads the group, starts accumulating excessive fat. When the light is turned off, the brothers’ activity period starts. It is getting quiet around them, they do not hear the humans anymore, just other mice from other homes nearby. This is a good time to take a meal and luckily, they do not need to search long for their food, there is enough for everyone. Apart from food, their cage is boring because there is nothing to explore and barely anything to play with or space to run around. This is why Blacky and his brothers groom each other a lot (**4**) (94) causing small bold patches, sometimes even wounds and infections (95, 96). The grooming follows a strict hierarchy, and Black-Jack gives all the calls. If another brother has a different opinion, Black-Jack is showing him who is the boss by biting him. These fights and dominance behaviors alter neuroendocrine mediators such as corticosterone and tyrosine hydroxylase (97). After feeding, grooming and fighting, there is nothing much to do and since the brothers were woken up several times during their rest period, this is a good time to sleep again (**5**) (98). Unfortunately, this sleep is never as good as when the lights are on but who knows when to be active and when to sleep anyways?

Laboratory mice do not have to scare predatory animals, but care takers and scientists are fulfilling this aspect sufficiently. It was shown in several experimental studies that handling and exchange or cleaning of cages is inducing stress in mice (92, 93), especially when male care takers and scientists are involved (99) (**Figure 2** and **Box 1**). The laboratory mice cannot entirely follow their circadian rhythm as most experimental interventions take place during the work hours of the scientists and animal caretakers, thus in the rest period of these animals during the day. It therefore cannot be excluded that laboratory mice are constantly sleep deprived and suffer somehow from “shift work disorder”. Keeping a mouse below its thermoneutral zone of 30°C ambient temperature may further activate the stress systems and interfere with circadian regulation (100), sleep and immunity (101). In most cases, studies in mice on the circadian system or sleep address these issues. In their experimental designs the light-dark cycle is changed on purpose (i.e., the dark period for the animals is during the daytime working hours of experimenters). Manipulations are in this way mainly scheduled to the active period of the animal and experimenters work in dim or red light that does not impact the SCN. As shown in **Figure 1**, mice in nature are exposed to circadian changes in ambient temperature, which are not present in laboratories, also leading to differences in sleep and circadian alignment (100, 102). However, the fact that warmer ambient temperature, the availability of nesting material and group housing support sleep in mice is taken into account in most laboratories (45).

Shift work can be mimicked experimentally in mice by changes in the light-dark schedule. As food intake is an important external time cue, time restricted feeding can be seen as another method to mimic shift work, in particular, when food is offered during the light phase, thus during the rest period of mice (103, 104). In *ad libitum* feeding protocols, food intake is often not controlled for, although it has a major impact on skin clocks (105) and on immune outcomes in response to circadian or sleep manipulations (106). Apart from changes in external time cues to mimic shift work, in animals the circadian system can be directly targeted by SCN lesions or genetic manipulations of clock genes in the germline or on a cell-specific level (e.g., *Clock* knock-out in particular immune cells, or knock-downs by adeno-associated viruses) (10, 107–110). Notably, these interventions might also induce sleep changes that should be controlled for (111). On the other hand, sleep can be manipulated by various more or less stressful techniques of sleep deprivation or fragmentation, or by optogenetics (112). However, it should be kept in mind, that mice cannot follow a constant routine to avoid confounding influences of physical activity during induced wakefulness. Mice are still the most common animal model although they are nocturnal animals with a complementary sleep-wake rhythm compared to humans. However, not all cellular or endocrine factors have complementary rhythms. Melatonin for example has a similar pattern in mice and humans, underlining its role as a dark-signal, which is downstream activating different pathways. Noteworthy, it is under debate for many mouse strains whether they are able to synthesize melatonin (113, 114). Despite this, melatonin was shown to affect mice regarding depressive-like and stress behavior and also circadian alignment (115–117).

On the other hand, mice offer plenty of possibilities to study sleep-wake behavior, e.g. by implanting electrodes or by assessing circadian locomotor activity patterns with electronic running wheels (even in group housed mice) (118). It is also possible to implant radiotelemetry transmitters to constantly measure the heart rate and blood pressure (119, 120) or using photobeam, and electroencephalograms. Metabolic feeding cages using indirect calorimetry are also a great option to follow the circadian metabolic patterns of mice (121). Genetic knock-outs, knock-downs and knock-ins make these animals a valuable tool to examine the effect of specific genes. It should be taken into account, however, that extended breeding, husbandry and genetic manipulation of lab mice resulted in profound changes in gene expression distancing them further from wild animals (89).

The murine immune system is only partly comparable to that of humans with various cellular and molecular differences (122, 123). Neutrophils display about 10-25% of the cells in the peripheral blood in mice whereas these are 50-70% in humans. Lymphocytes are the most abundant cell type in peripheral blood in mice with 75-90%, compared to only 30-50% in humans (122). Mice are also commonly used to assess skin biology. This is an interesting option to induce certain diseases such as epidermolysis bullosa acquisita (EBA) (124–126), and allows to take skin and organ biopsies in a degree that is not possible to gain from human patients. Human and murine skin are composed of the same layers. However, the thickness of human skin is much higher and more adherent to underlying tissues. Accordingly, mice have decreased barrier function and enhanced percutaneous absorption, which should be considered when using murine models for topic drug delivery. Moreover, mice have fur and therefore more hair follicles, which leads to differences in wound healing (127, 128). In contrast to humans, mice also show a subcutaneous layer called *panniculus carnosus*, which is a muscle layer, enabling skin contraction. This is of interest since large wounds require muscle contraction for healing, whereas in humans wound healing is achieved by formation of granulation tissue and reepithelization (129).

Even though rodents have the above-mentioned limitations, they are nevertheless a valuable and hitherto irreplaceable tool for studying effects of sleep and the circadian machinery on the immune system. Murine studies allow to explore the relations and connections between different organs and influences of the lifestyle, which cannot be shown in other models. This is either due to limitations in genetic manipulations in non-rodents or because the model is not a full organism such as cell- or organ-cultures. Hopefully, future techniques are overcoming the need for murine studies and thereby offer new models without the above-mentioned drawbacks of mice. It is important to address difficulties with certain models to be able to overcome these. Nevertheless, there are various well-working mouse models, which delivered valuable results. Just to mention two out of many studies, we would like to outline the work of Toth et al. who studied the effects of shift work in a lupus mouse model, which resembled also typical human outcomes (130) as well as the work of Sadeghi et al. who used an EBA mouse model with an unbiased genetic approach to investigate inflammatory processes and discovered the role of the clock gene retinoic acid-related orphan receptor-alpha (*Rora)* in this disease (126).

Apart from invertebrates that cannot be used to assess skin diseases, other animal models are fish to study melanoma (131, 132) or mammals such as pigs to study skin and circadian regulation of the immune system (133, 134). However, porcine models are difficult to establish as the animals need a lot of space and require comparably long breeding spans, although some genetical modifications such as CRISPR/Cas are already well-established (135). Regarding non-human primates, pigs are ethically more accepted for experimental purposes and show higher numbers of offspring, allowing to gain sufficient animals for statistical analysis of the experiments.

## 4 Epidemiological and experimental evidence that shift work could promote skin autoimmune diseases

This section outlines the connection between shift work and skin autoimmune diseases. Firstly, described by shift works’ general effect on the immune system and then depicting more detailed effects on certain skin autoimmune diseases.

### 4.1 Shift work induces systemic chronic inflammation, immunodeficiency, and dysregulation of adaptive immunity

Shift work drives SCI (25, 136, 137) that is associated with endothelial dysfunction, atherosclerosis, cardiovascular diseases, impaired glucose tolerance, metabolic syndrome, diabetes mellitus, obesity, mood disorders and neurodegenerative diseases (22, 24, 136, 138). Likewise, experimental circadian disruption as well as sleep deprivation in humans and animal models can induce an inflammatory response (40, 139, 140) and dysfunctions of cardiovascular processes (75, 141, 142), metabolism (143, 144), mood and cognition (145). Presumably, SCI in the periphery and a parallel neuroinflammatory response in the brain are the consequence of innate immune cells responding to sterile immune stimuli (e.g., reactive oxygen species, metabolites, danger/damage associated molecular patterns) (37, 146–148) and failures in counter-regulatory, anti-inflammatory mechanisms that normally regulate and resolve inflammation (e.g., IL-10, resolvins, M2 macrophages, regulatory T cells (Tregs)) (47, 149–151). While the innate immune system fights this unnecessary battle against sterile stimuli, targeted and protective immune responses are compromised. Thus, shift work or experimental circadian disruption, as well as insufficient sleep are associated with failures in innate and adaptive immunity against pathogens (152) and tumors (153–156) and with reduced vaccination-driven T cell and antibody responses (157–160), both in humans and in mice. On the other hand, shift work seems to boost unwanted adaptive immune responses to harmless antigens such as allergens and auto-antigens. In detail, shift work, circadian misalignment, clock gene polymorphisms or poor sleep are associated with a higher risk to develop allergic or autoimmune diseases (31, 161). The latter include (i) connective tissue diseases such as systemic lupus erythematosus (SLE) (162), systemic sclerosis (SSc) (161), and Sjögren’s syndrome (163, 164), (ii) different forms of arthritis such as rheumatoid arthritis (RA) (165), and spondyloarthritis (SpA) (161, 166) including psoriasis arthritis (163), (iii) inflammatory bowel disease (IBD) (167–173), (iv) autoimmune thyroiditis (32, 174–176), and (v) multiple sclerosis (177). Undoubtedly, sleep is impaired in patients with systemic autoimmune diseases (178). Moreover, they show disturbances in 24 h rhythms of their stress systems (179, 180), cardiovascular functions (181), and melatonin (182–185). These changes may be the consequence rather than the cause of the autoimmune disease, as disease symptoms such as pain, itch, respiratory or gastrointestinal dysfunctions can heavily interfere with a regular sleep-wake behavior (186). However, lupus-prone mice show disturbed 24 h rhythms of corticosterone and melatonin already in asymptomatic phases, thus before manifestation of the disease (187). Moreover, experimental circadian disruption and/or sleep deprivation in animal models can promote autoimmune diseases like lupus (130, 188). It can also worsen colonic inflammation in murine models of IBD (189) or attenuate others such as experimental auto-immune encephalomyelitis (190). Thus, autoimmune processes and wake-sleep-disturbances most likely show bidirectional relationships that could feed into a vicious circle.

### 4.2 Does shift work boost skin manifestations of systemic autoimmune diseases or skin-specific autoimmune diseases?

#### 4.2.1 Systemic autoimmune diseases and thyroiditis

Many systemic autoimmune diseases affect the barrier organs, thus the mucosa of the respiratory, gastrointestinal, or urogenital tract or the epidermis, dermis and subcutis of the skin (**Table 2**) (191–260). Cutaneous manifestations are leading symptoms in SSc (e.g., puffy fingers, skin fibrosis and calcinosis cutis), or characteristic clinical presentations of SLE (e.g., malar ‘butterfly’ rash), of Sjögren’s syndrome [e.g., dry skin called xerosis cutis or xeroderma (232, 233)], of RA [e.g., rheumatoid nodules (241)], of SpA, celiac disease or IBD (e.g., pyoderma gangrenosum) (242, 252), or of thyroiditis [e.g., myxedema (259)]. Connective tissue diseases, but also any other systemic autoimmune disease can lead to secondary Raynaud’s phenomenon with an exaggerated cold-induced vasoconstriction of arteriovenous anastomoses in distal skin regions (e.g., fingers and toes). Currently, there is only limited data on the impact of the sleep-wake cycle on cutaneous symptoms of systemic autoimmune diseases or thyroiditis. The circadian system may be involved in photosensitivity in SLE (218). Moreover, poor sleep correlates with enhanced skin thickness in SSc (225, 227) and with genital ulcers in the vasculitis Behçet’s disease (235, 236, 261). However, although shift work has been shown to impair skin health with respect to allergic and cancerous conditions (262, 263), to our knowledge epidemiological or experimental studies on skin manifestations of systemic autoimmune diseases are currently lacking. Even for Raynaud’s phenomenon that involves a pathophysiology closely linked to circadian and sleep-dependent thermoregulation (which could be easily monitored by wearables), we did not find a single study that investigated this condition in shift workers.

**Table 2:** Effects of shift work, circadian or sleep disturbances on autoimmune (skin) diseases.

Disease	Skin manifestations	Findings in shift workers	Circadian findings	Sleep findings	Key cellular players	Involved neuroendocrine mediators
Skin-specific autoimmune diseases						
Psoriasis	Erythematosquamous plaques; rarely pustular changes; nail psoriasis in 50% of patients	Higher risk of development (191)	Generally disturbed 24 h rhythm (192); rhythmic regulation of neutrophil traffic (193); symptoms show diurnal pattern (163); symptom severity peaks in the evening and at night (194)	Pruritus disrupts sleep (195); systemic increase of proinflammatory cytokines by sleep deprivation in mice (196); increased frequency of sleep disturbances (197) and fatigue (198)	Neutrophils (193); gamma-delta T cells (199)	Dysfunctional HPA-axis with reduced cortisol levels; prolactin levels tend to be higher in psoriasis than in controls (200, 201); disturbed circadian rhythm of melatonin (202)
Vitiligo	Whitening due to destruction of melanin (203)	No data available	Blood rhythms in NK cell activity are shifted (204, 205), CD4 T cell number rhythm disrupted (206)	Sleep disturbances; poor sleepers show a higher risk of vitiligo (207, 208)	NK cells, CD4 T cells (204, 205)	Stress mediators and melatonin (209)
Pemphigus	Blisters in epidermis and mucous membranes; positive Nikolsky sign (210)	No data available	No data available	Bidirectional relationship (211)	Autoreactive B cells (212)	Corticosteroids are used as common symptomatic treatment; stress is able to trigger phemphigus flares and can worsen symptoms (213)
Pemphigoid diseases	Subepidermal blisters; negative Nikolsky sign (210)	No data available	No data available	Sleep disturbances due to symptoms peaking at night (66, 67, 214)	Autoreactive B cells; neutrophils (124, 215, 216)	Corticosteroids are used as common symptomatic treatment (217)
Systemic autoimmune diseases						
Connective tissue diseases						
Systemic lupus erythematosus	Malar rash, photosensitivity, alopecia, livedo reticularis (218)	Higher risk of development (162)	Photosensitivity (218); symptoms show diurnal pattern (163); excessive daytime-fatigue, especially in the morning (219)	Sleep disorders increase the risk of disease development (161)	B cells, plasma cells, Tregs (220); oxidative stress caused by Th17 cells (221)	Dysfunctional HPA-axis with reduced cortisol levels and changes in prolactin (222) and melatonin (221, 223); adrenal insuffiency (224)
Systemic Sclerosis	Puffy fingers, skin fibrosis, skin ulcers, calcinosis cutis, teleangiektasia (225)	No data available	Altered prolactin rhythms (226); symptoms show diurnal pattern (163)	Poor sleep leads to enhanced skin thickness (227); therapeutic sleep can be used to ameliorate skin symptoms (228); fatigue (225)	Th2 cells, B cells, macrophages (229)	Lower cortisol (230) and melatonin levels (231); altered prolactin rhythm (226)
Sjögren’s syndrome	Xeroderma (232)	Higher risk of development (163)	Circadian disruption is enhancing disease onset and progression (164); symptoms show diurnal pattern (163); excessive daytime-fatigue (219)	Patients often suffer from excessive daytime sleepiness, fatigue, insomnia, nocturnal headaches and nocturnal sweats (178); sleep disorders increase the risk of disease development (161)	Memory B cells, marginal zone B cells, plasma blasts and plasma cells (233)	Hypofunctional HPA-axis resulting in lower basal ACTH and cortisol levels (234)
**Vasculitis**	Purpura; Behcets syndrome (235, 236)	No data available	Inadequate decrease of nocturnal blood pressure (237)	Fatigue (238)	Dendiritc cells, Th17 cells and macrophages (239) and B cells (240)	Melatonin is able to relieve vascular endothelial cell damage (185)
Arthritis						
Rheumatoid arthritis	Rheumatoid nodules, Felty syndrome, rheumatoid vasculitis, pyoderma gangrenosum, rheumatoid neutrophilic dermatosis, interstitial granulomatous dermatitis, palisaded neutrophilic dermatitis (241)	Higher risk of development (161); independency of higher risk for development from socioeconomic factors, health behavior or psychological distress (243); development of sero-positive rheumatoid arthritis was especially increased in rotating shift work and day-oriented shift work, whereas increasing duration of permanent night shift appears to be protective against rheumatoid arthritis (33)	Symptoms show diurnal pattern (163); peak of symptoms between 02 and 04 AM (244); excessive daytime-fatigue, especially in the morning (219); Disruption of the circadian clock results in aberrant expression of inflammatory cytokines (e.g. IL-6) disruptions of the peripheral chondrocyte clock promotes catabolic processes in cartilage (245)	Sleep disorders increase the risk of disease development (161); fatigue as common symptom (163); sleep disturbances, poor sleep quality and decreased total sleep time are common in rheumatoid arthritis; short sleep duration is causally linked to an increased disease risk (165)	T cells (Th1, Th2, Th17, Treg), cells of the B cell compartment, macrophages (246)	Dysfunctional HPA-axis and altered circadian rhythm of cortisol (248) and melatonin (183)
Spondyloarthritis	Pyoderma gangrenosum, hidradenitis suppurativa (242)	Higher risk of development (161)	Symptoms show diurnal pattern (163); major peaks in the morning between 06 AM and 09 AM (166)	Sleep disorders increase the risk of disease development (161); disease is also by sleep disturbances (178)	IL-17 is a key mediator which is produced by a variety of cells such as Th17 cells, neutrophils and macrophages (247)	Increase in backpain at midnight might be related to lower melatonin levels in spondyloarthritis patients compare to healthy controls (249)
**Inflammatory bowel disease**	Pyoderma gangrenosum, hidradenitis suppurativa (242)	Higher risk of development (167, 168); risk factor for surgery in Crohn’s disease, but not in ulcerative colitis (172); extended and irregular shift work might be a risk for chronic inflammatory bowel disease (173)	Disruption of the circadian system increases the activity of the gut immune system and the release of inflammatory factors; diurnal oscillations of microbiota (170)	Sleep disturbances are risk factors for development of Crohn’s disease in children (171); increased risk for ulcerative colitis in people sleeping less than 6 h and more than 9 h per day (173)	(169) Crohn’s disease: Th2; ulcerative colitis: Th1; both: Th17, Treg (250)	Lower 24 h amplitude of plasma cortisol in ulcerative colitis (251), lower levels of melatonin in ulcerative colitis (184)
**Celiac disease**	Dermatitis herpetiformis; association to psoriasis, chronic urticaria, leukocytoclastic vasculitis, alopecia areata (252)	No data available	No data available	Data on the presence of insomnia and sleep disturbances in patients with celiac disease is heterogeneous (253–255). sleep disturbances as well as primary sleep disorders such as sleep apnoea improve under gluten-free diet (256, 257)	Th1 cells, cells of the B cell compartment (258)	No data on the role of neuroendocrine mediators in dermatitis herpetiformis
**Thyroiditis**	Myxedema (259)	Higher risk of development (32)	Downregulated *BMAL1* and *PER2* expression (175)	Often occurs together with obstructive sleep apnoea but unclear if cause or consequence (176)	Infiltration of T cells, which release inflammatory cytokines (260)	Significant decrease of serum melatonin levels (175)

Overview of autoimmune diseases and the effects of shift work and the circadian rhythm. Abbreviations in order of apearance: HPA, Hypothalamus-pituitary-adrenal; NK, natural killer; Th, T helper; Tregs, regulatory T cells; ACTH, adrenocorticotropic hormone; ACPA, anti-citrullinated protein antibody; IL, interleukin; UC, ulcerative colitis No relevant results were available for mixed connective tissue disease, polymyositis or dermatomyositis.

#### 4.2.2 Psoriasis

Likewise, studies on the association between shift work, circadian disruption or poor sleep on skin-specific autoimmune diseases are rare and mainly focus on psoriasis. Psoriasis typically presents with cutaneous erythematosquamous plaques and approximately 50% of patients develop typical nail changes. Rarely, pustular changes occur, which can affect the palms and soles, but also the entire body. One aspect of psoriasis research that has recently been investigated is the time-of-day variability in disease symptoms and severity with a peak of itch and psoriasis flares in the evening and at night. However, the reasons for this observation remain unclear (194). Related diseases such as SpA show similar peaks of symptoms at night (249).

Early circadian research on psoriasis investigated time-of-day-dependent changes in the epidermis. One study revealed no diurnal differences in mitotic index (264), whereas another one showed increased cell proliferation at 6 AM compared to healthy controls (265). A further study investigating circadian cell kinetics revealed a stable epidermal and dermal infiltrate cell proliferation over the day both in uninvolved and involved psoriatic skin, but with a circadian rhythm in epidermal DNA synthesis (266). However, in 1985, a 24 h rhythm of neutrophil migration in psoriatic skin with a peak at around 10 PM was detected that could not be shown in the skin of healthy controls (193). Further reports suggest a systemic circadian perturbation, with disruption of circadian rhythms of urinary and haema-tological parameters (192), of blood pressure and heart rate (237, 267) and of plasma melatonin levels (202) in psoriasis (and vasculitis) patients. In addition, lower urinary 24 h cortisol levels, lower serum cortisol at 8 AM and 5 PM were found in patients with psoriasis and IBD (251) compared to healthy controls suggesting an altered function of the HPA-axis (268).

To unravel the link between the circadian clock and psoriasis, a transcriptomic study showed a downregulation of *CRY1/2, REVERBA, CLOCK, BMAL1* and *RORA/C* in keratinocytes from psoriatic lesions (269). A recent study found changes in core clock genes and clock proteins in non-lesional and lesional human skin samples in psoriasis (270). In a mouse model, imiquimod-induced psoriasis-like dermatitis was ameliorated in mice with a loss of function mutation in the *Clock* gene compared to wild-type mice. Accordingly, in mice with a loss of function mutation in the *Per2* gene imiquimod-induced psoriasis-like dermatitis was exaggerated because PER2 inhibits CLOCK activity (271).

Based on the described investigations, few groups studied the application of chronotherapy in psoriasis. Balneotherapy (bathing therapy) has the highest efficiency when it is applied in the morning (272). Topical corticosteroid application in the evening has a higher efficiency than application in the morning after two days of treatment. However, this difference evened out after five treatment days (273). Discussed reasons for the higher effectiveness are an improved corticosteroid absorption in the evening due to a higher cutaneous perfusion and a higher skin barrier permeability or a higher therapeutic potential due to a rise in inflammation and cell proliferation in the skin during evening hours (273, 274). Recently, chronotherapy of maxa-calcitol, a vitamin D analogue, was investigated in a mouse model of psoriasis in which the skin inflammation was induced by topical 12-O-tetradecanoylphorbol-13-acetate (TPA). In the skin of mice, expression of the nuclear vitamin D receptor exhibits a distinct daily variation with a peak in the middle of the active period. Accordingly, in TPA-mice application of maxacalcitol during early to middle of the active period had the highest therapeutic efficacy (275).

Various studies describe the occurrence of sleep disturbances and fatigue in patients with SLE, Sjögren’s syndrome, vasculitis, psoriasis, and psoriatic arthritis (197, 198, 219, 238, 276–279). In both conditions, patients rate sleep disturbances as a factor severely impairing quality of life (280–283). Sleep disturbances in patients with psoriasis and psoriatic arthritis are caused not only by disease manifestations such as nocturnal itching or pain (20, 284), but also by gastroesophageal reflux disease, anxiety or depression (285, 286). In addition, an association between psoriasis and prevalence of sleep disorders, such as obstructive sleep apnea or restless legs syndrome, has been described. Data on the prevalence of sleep disorders in psoriasis and potential effects of psoriasis treatment with biologics on sleep and sleep disorders are presented in a recently published review (287). Until now, the effect of immunomodulatory and immuno-suppressive therapeutics on sleep disturbances remains elusive (288–290). In addition, studies investigating the relationship between sleep quality and disease activity in psoriasis and psoriatic arthritis give contradictory results (276, 291–298). So far, studies on lifestyle interventions that affect sleep and investigate respective effects on disease activity are lacking (299). Impairment of sleep in patients with psoriasis, psoriatic arthritis and axial SpA increases the risk for psychiatric diseases, which themselves might impair sleep, resulting in a vicious circle (300–302). Moreover, patients with psoriasis and sleep disturbances have a higher risk of stroke and ischemic heart disease compared to psoriatic patients without sleep disturbances (303). Finally, jet-lag in patients with psoriasis experiencing a flight crossing at least two time-zones increases self-reported disease severity (304).

Circadian and diurnal variations as well as sleep dis-turbances are well investigated in psoriasis and psoriatic arthritis but only a single study investigated the influence of shift work on the risk of psoriasis. Li and colleagues published a study in 2013 showing that enhanced duration of rotating night shift work increases the risk of psoriasis independently of important behavioral risk factors for psoriasis, namely body mass index and smoking (191). Moreover, night shift work is associated with an increased risk of psoriasis comorbidities, e.g. myocardial infarction (305).

#### 4.2.3 Other skin-specific autoimmune diseases

Apart from psoriasis further skin-specific autoimmune diseases are vitiligo, pemphigus, EBA, and bullous pemphigoid (BP). Poor sleepers show a higher risk of vitiligo (306, 307). There are also reports on sleep disturbances in patients with BP (67) or vitiligo (207, 208). For EBA, the clock gene *Rora* was found to be a genetic risk locus in the murine passive anti-collagen type VII transfer model (126). The core-loop of the circadian clock consists of *Bmal1/Clock* and *Per/Cry*. However, more genes interact and form additional loops. The transcription of this clock gene *Rora* is initiated by *Bmal1/Clock*, which itself fosters the transcription of *Bmal1* (308). Sadeghi and colleagues found that a knockout of *Rora* in mice diminished the skin lesions upon anti-COL7 challenge. They could further show that even a *Rora* blockade was able to reduce skin inflammation and blistering in this model (126). Patients with BP (mostly elderly) are often also diagnosed with neuro-psychiatric comorbidities years before skin manifestations of BP appear (309). It turned out that anti-BP230 (one of the autoantibodies emerging in BP), is an independent predictor of neuro-psychiatric illnesses in BP patients (310). In addition to the dermis, BP230 is also expressed in the central nervous system and an immunologic cross-reaction of the autoantibodies causing neuroinflammation might explain the BP-associated neuropsychiatric disorders (311). Circadian disruption (e.g by aging or sleep disturbance) increases neuroinflammation in rats (312, 313) and humans (24, 314) and might amplify neuropsychiatric symptoms in BP patients. If this scenario is translatable to shift work remains to be elucidated. Symptoms of autoimmune skin diseases peak at night, as shown by actigraphy also in BP patients (66, 67). This could further disrupt sleep and circadian rhythms thus feeding into a vicious cycle.

Apart from autoimmune skin diseases, disturbances of the wake-sleep cycle seem to promote also infectious skin diseases such as bacterial invasions (315), allergic skin diseases such as atopic dermatitis (316), contact hypersensitivity (317), or skin cancers (318).

In general, skin diseases show distinct 24 h rhythms in symptoms (163), with a nocturnal peak in pain, pruritus and scratching that heavily interferes with sleep (195, 244, 319–321). The dermatology life quality index (DLQI) questionnaire is not considering sleep disturbances (322), whereas the bullous pemphigoid disease area index (BPDAI) explicitly queries sleep impairments to quantify the extend of pruritus (217, 323). These questionnaires are not standardized to span different (autoimmune) skin diseases, which renders it difficult to compare them with each other. The Pittsburg Sleep Quality Index (PSQI) covers various aspects of sleep, its duration and disruptions but does not include any aspects of autoimmunity besides unspecified pain at night (324). Sleep disturbances due to nocturnal symptoms should therefore more often be rated in clinical scores of skin diseases or by objective assessments using, e.g., wearables (66, 67).

## 5 Potential effects of shift work on skin and immune cells

So far, we described the manifold influences of the circadian system on sleep, behavior, thermoregulation, neuroendocrine mediators, the immune system, skin manifestations of systemic autoimmune diseases and skin-specific autoimmune diseases. In the following, we will outline how the circadian system and the sleep-wake cycle physiologically regulate skin and immune cells and how disturbances of this regulation, e.g. due to shift work, could drive the pathophysiology of these disorders.

### 5.1 Potential effects of shift work on skin cells

**Figure 3:**
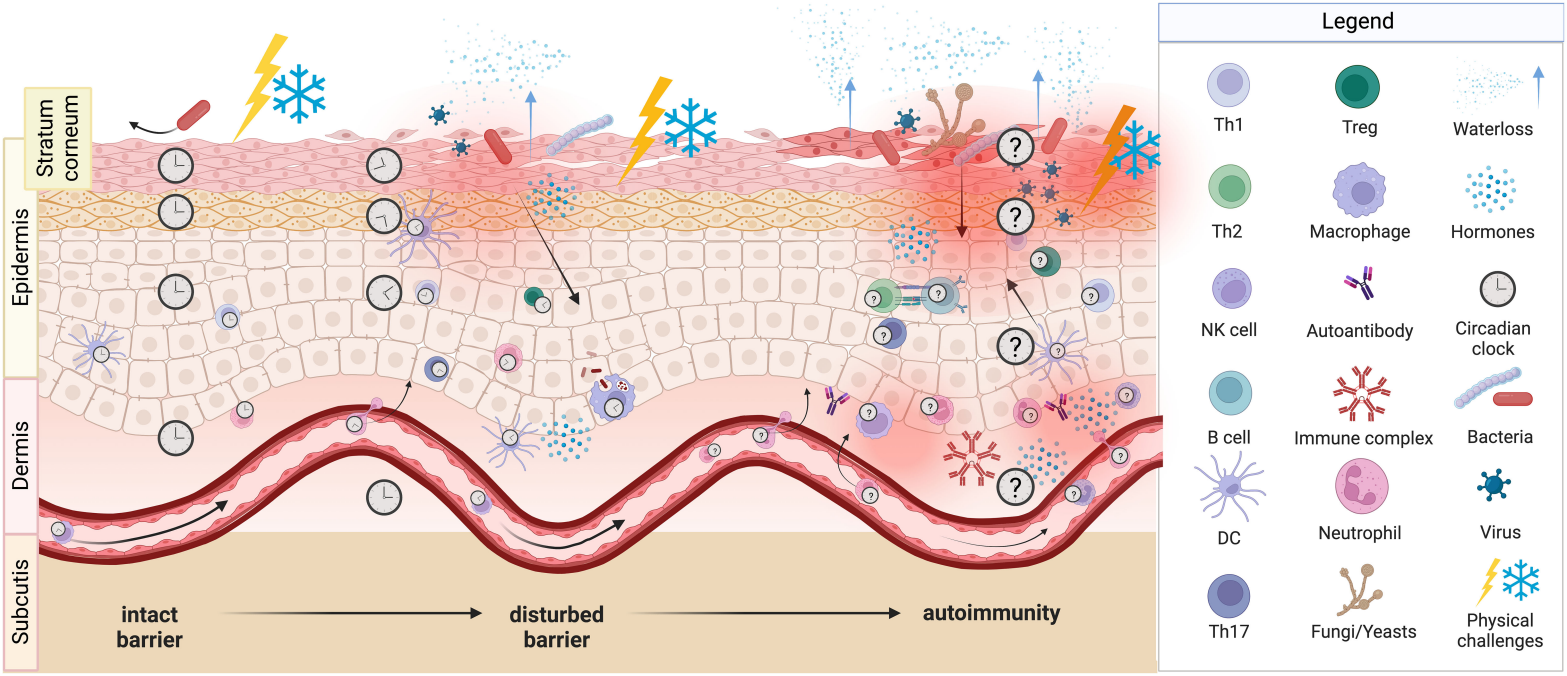
Potential consequences of shift work. Circadian misalignment and/or sleep impairments as a result of shift work presumably lead to changes in the skin barrier function. An intact barrier (left part) is able to block physical challenges as well as pathogens and prevents transepidermal water loss, whereas a disturbed barrier (middle part) is not able to do so. Noxi and intruders then can reach the epidermis, induce damage and activate immune cells and thus local inflammation can occur. A severely disturbed barrier (right part) shows breaches, through which bacteria, viruses and fungi enter the skin and cause inflammation with the attraction of various innate and adaptive immune cells. Likely, consequences are itching, scratching, and pain. Molecular clocks tick in skin cells, in innate and adaptive immune cells, as well as in endothelial cells and could be entrained by light, temperature, and neuroendocrine mediators such as cortisol, catecholamines and melatonin. We hypothesize, that this circadian system of the skin strengthens barrier functions during daytime and that shift work-induced changes favor the development of autoreactive T cells and autoantibodies resulting in autoimmune diseases.

It is hypothesized that the circadian system evolved to protect proliferating cells from DNA damage due to UV light (10, 325, 326). Such a rhythmic adaptation to light or to other environmental stimuli like ambient temperature, moisture, or pathogens conceivably is most relevant in the skin, the major barrier between the outer and the inner world (**Figure 3**). Thus, multiple epidermal, dermal and hair follicle clocks tick in stem cells, keratinocytes, fibroblasts, and melanocytes and jointly seem to serve protection against environmental challenges during the activity period (49, 109, 327–332). Notably, clock genes also regulate cellular functions in human keratinocyte and melanoma cell lines (327). The subcutis with its fat depots and its dense sympathetic innervation and vascularization also seems to have a circadian regulation that might serve in particular metabolic and thermoregulatory functions (333). Presumably, this skin clockwork maintains skin physiology and skin integrity (39, 331). Thus, skin blood flow, temperature, pH, transepidermal water loss, and sebum excretion show 24 h rhythms in humans (334–336) and animals (337). The circadian system also impacts wound healing in mice (338) and hamsters (339). Experimental disruptions of this finetuned rhythmic skin regulation by circadian misalignment or sleep deprivation impairs skin integrity (65, 340), regeneration (341, 342), and wound healing (339, 343, 344), accelerates skin aging (345), enhances the activity of skin proteases (196), and leads to skin ulcers and hyperkeratosis (346). These outcomes prompt symptoms such as itching and pain. Interestingly, the pheno-menon that pruritus and scratching peak at night, is presumably driven by the described rhythmic changes in skin barrier functions and by enhanced nocturnal skin temperature (214). Although pain perception shows conflicting results with respect to circadian regulation in humans (347, 348), it clearly increases upon circadian or sleep disturbances (349). Vice versa, as outlined above itching and pain at night are likely explanations of sleep disturbances in patients with skin diseases (277, 319). Thus, circadian disruption, sleep loss, enhanced scratching, and exaggerated pain perception likely feed into a vicious circle that further fosters skin barrier damage. Shift workers show disrupted rhythms in hair follicle cells and interfollicular epidermal cells (341, 350) and changes in pain perception (351). However, it is presently unknown, whether shift workers suffer from impairments of skin physiology or integrity. Itching leads to scratching, causing wounds that need to heal. In mice, wounds occurring during the rest phase healed less quickly than wounds that occurred during the active phase. Responsible for this is the rhythmic mobilization of fibroblasts by dynamic actin (338). Similarly, it was found in mice that sleep fragmentation delays wound healing (352). Likewise, difficulties in wound healing are commonly observed in patients with autoimmune diseases and sleep abnormalities (353).

### 5.2 Potential effects of shift work on innate immune cells in the skin

The skin is populated by transient and resident innate immune cells. Previous studies showed a sleep-wake cycle-dependent hematopoietic release of granulocytes and monocytes and subsequent traffic of these cells to various tissues (110, 354–356). Although rhythmic leukocyte homing to skin seems to be neglectable in the steady state (355), indirect evidence indicates that there is a circadian regulation of immune cell traffic to the skin upon wounding and upon microbial or antigenic challenges (357–362). Likewise, tissue-resident innate immune cells like dendritic cells (363, 364), mast cells (317, 365, 366) or macrophages (367–369) show circadian regulation that impacts cutaneous responses to antigens and allergens, respectively. Several genes controlling immune functions are rhythmically regulated in murine skin (331) and the expression of cytokines (e.g. TNF) and chemokines (e.g. IL-8 or C-X-C-motif ligand 1) in human skin are under clock control as well (370). Overall, disturbances of these fine-tuned rhythms in leukocyte traffic and function seem to result in unchecked innate immunity. This is a mechanism that could also contribute to systemic inflammatory responses with increases in blood leukocytes, neutrophils, monocytes, and C-reactive protein in shift workers (75, 136, 138, 371–376). Along this line, both experimental circadian disruption and sleep deprivation enhance the responsiveness of the innate immune system to inflammatory stimuli and trigger inflammation (75, 106, 139, 354, 356). The outlined interactions between the sleep-wake cycle and innate immunity may be relevant for skin diseases, as sleep deprivation induces systemic increases in pro-inflammatory cytokines also in a mouse model of psoriasis (196), and as neutrophil traffic into the skin of psoriatic patients shows rhythmic regulation (193). Apart from granulocytes, monocytes, dendritic cells, and mast cells, also natural killer (NK) cells show 24 h rhythms of their numbers and their activity in human blood (377–379) and rodent spleen (154, 380). Circadian and sleep manipulations alter these parameters (154, 340, 377, 378, 380–382). Likewise, experimentally simulated shift work in healthy individuals changed gene transcripts of NK cell-mediated immune responses (77) and shift workers show impaired NK cell-function (383, 384). Also, in patients with systemic autoimmune diseases (385) or with vitiligo (204, 205), altered rhythms of NK cell-activity in peripheral blood were described. Time-of-day dependent changes in NK cell numbers or functions in healthy or diseased skin, however, were not tested so far.

### 5.3 Potential effects of shift work on adaptive immune cells in the skin

T cells play key roles in a variety of autoimmune diseases (239, 240, 247, 250, 258, 260) and can enter and reside in the epidermis and dermis. Their recirculation between blood, lymphoid organs and other tissues is regulated by the circadian system (190, 382, 386) and by sleep (387, 388). This is presumably mediated by sleep-wake cycle dependent changes in T cell selectins, integrins, and chemokine receptors, and in corresponding ligands on endothelial cells and surrounding tissues (190, 386, 389, 390). Moreover, T cell functions like proliferation (151, 391), Th1-, Th2-, and Th17-differentiation or cytokine production (392–398), and the activity of Tregs (151, 399, 400) are linked to the sleep-wake cycle. Rhythmic changes in T cell traffic and functions seem to be regulated by T cell intrinsic clocks such as *REVERBA* (398, 400, 401) and by effects of the SCN or sleep on neuroendocrine mediators (see next section) (387, 390, 402). In shift workers, increases in T cell numbers (384, 403) and impairments in T cell proliferation (28) were reported.

Joint effects of the circadian system and sleep on T cell immunity could also contribute to sleep-wake cycle dependent changes in cutaneous T cell responses and disturbances thereof in shift workers. Indeed, the T cell driven induction or recall of cutaneous DTH reactions show rhythmic modulation in humans (404, 405) and rats (406, 407). Primary DTH responses were impaired in stroke patients showing sleep rhythm disturbances (408) or in hamsters upon experimental circadian disruption (363) or light at night (409). In contrast, *Clock* mutant mice showed enhanced T cell driven contact hypersensitivity to allergens upon challenge (317). Likewise, constant light in mice enhanced allergic skin responses, while the development of immune tolerance and subsequent Treg infiltration in the challenged skin was impaired (410). Another mouse experiment demonstrated reductions in skin allograft rejection and in T cell infiltration of the graft by sleep deprivation (411). Overall, the picture suggests, that dis-turbances of the circadian system and/or sleep could impair developing but exaggerate established T cell responses in the skin. With respect to skin autoimmune diseases, one study reported disrupted rhythms in blood CD4 T cell numbers in patients with vitiligo (206). In mouse models of psoriasis, clock gene mutations changed skin inflammation by modulating IL-23 receptor expression in gamma delta T cells and subsequent IL-17 and IL-22 production (271) and treatment with a REVERB agonist suppressed IL-17 production in gamma-delta T cells and improved dermatitis (199). In humans, clock genes might likewise impact cutaneous T cell responses, although this was so far only elaborated in the context of skin cancer (412).

Clocks also tick in B cells of mice (413) and humans. There are 24 h rhythms in human B cell numbers in blood (414) and murine B cell numbers in spleens and lymph nodes (415), and in systemic levels of antibodies, so called immunoglobulins (Ig) of the three subtypes IgG, IgA, and IgM (416–418). In line with exaggerated allergic DTH responses that are driven by T cells, *Clock* mutant mice also show enhanced IgE reactions to allergens (317). There is evidence that day-night-shift rotations attenuate the release of the anti-inflammatory cytokine IL-10 by B cells (419). This lack of immunologic regulation could be deleterious as the IL-10 releasing B cells of shift workers were unable to inhibit the proliferation of T cells (419). It could also be shown that *CLOCK* expression in peripheral B cells of shift workers was higher, which leads to a reduced expression of transforming growth factor beta (a cytokine mainly released by Tregs) (420). *Cry 1/2* deficient mice showed an autoimmune phenotype with elevated levels of serum IgG, antinuclear antibodies (ANAs), and immune complexes, as *Cry* presumably regulates B cell development and B cell receptor signaling (421). On the other hand, the distribution of B cell subsets in spleen, lymph nodes and peritoneal cavity in wild type and *Bmal1* knockout mice did not differ. The maturation of B cells was also not influenced by the knockout of *Bmal1*. Moreover, there was minor circadian regulation of *Per2*, which was detected by a reporter mouse model. It seemed as if cell intrinsic circadian clocks did not affect the B cells. They were probably gated by cell-extrinsic circadian variations (422).

B cells generate long-lasting immunologic memory by becoming (auto-)antibody-producing plasma cells and being able to survive decades in niches in the bone marrow (423). Therefore, plasma cells can be key in autoimmunity. In several cutaneous autoimmune diseases, auto-antibodies are a common diagnostic criterion and B cells are also one of the most often targeted cell types in the treatment of autoimmune diseases (424). In accordance, disease severity of pemphigus, BP, and SSc correlated with the number of B cells infiltrating the skin (210, 212, 215, 216, 229, 425). Auto-antibodies can be developed against all kinds of self-molecules. For connective tissue diseases such as SLE and SSc, for example, ANAs are formed against contents of the cell nucleus (220). This content is presumably presented for extended time to the immune system due to insufficient clearance after cell death (426). Apoptosis is a natural process but can also be triggered by UV light, explaining the photosensitivity in SLE (218).

Plasma cells cannot only develop in primary and secondary lymphoid organs by the help of T cells but also in a T cell-independent manner in the skin. This phenomenon was observed in several autoimmune and inflammatory diseases and the effect of local auto-antibody secretion is believed to play a role in chronic inflammation (424). Unfortunately, the influence of the circadian system or sleep on these skin-resident B cells is currently unknown.

## 6 Candidate neuroendocrine mediators linking shift work with skin autoimmune diseases

This section concentrates on central and peripheral hormonal agents that connect circadian, neuronal, and immunologic mechanisms.

**Figure 4:**
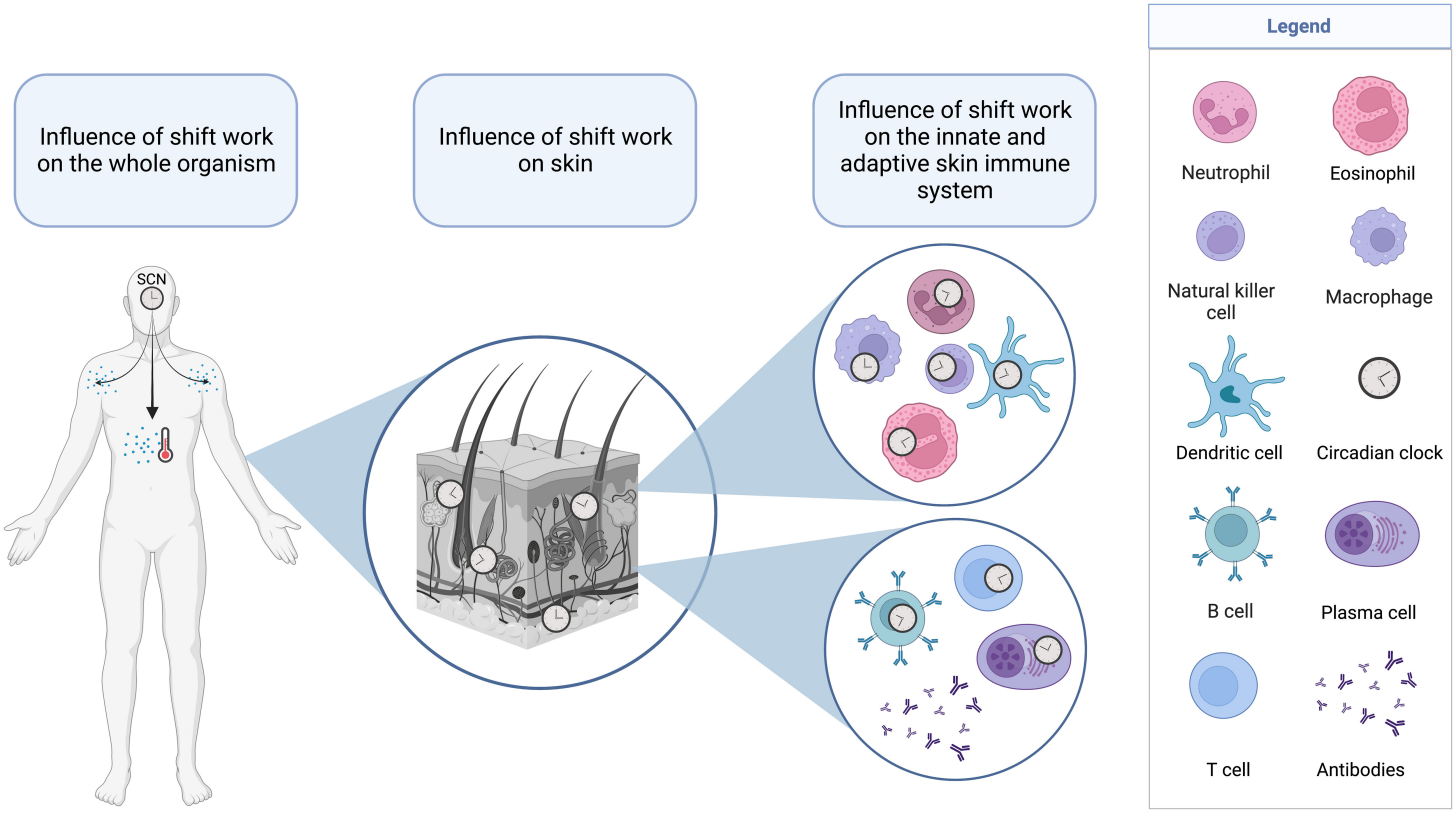
Layers of systems in the human body that are affected by shift work. Circadian disruptions due to shift work can affect the suprachiasmatic nuclei (SCN) and the major outputs of this master clock such as core body temperature and neuroendocrine mediators. These systemic influences as well as direct effects of ambient temperature and light can affect the circadian system in the skin and the cellular clocks of skin leukocytes of the innate (top) and adaptive (bottom) immune system.

The exact contributions of the SCN (109) with their systemic signals (e.g., stress mediators or core body temperature), of external time cues that directly affect the skin (e.g., extraretinal photoreception by keratinocytes, changes in ambient temperature) (427, 428) or of sleep and associated changes in behavior (e.g., darkness, supine position, reduced physical activity, fasting) (333, 390) in the entrainment of skin clocks and in the circadian regulation of the skin remain to be elucidated (429) (**Figure 4**). Whatever the case, the stress systems and melatonin seem to be key, either as SCN outputs to the periphery, as signals in cutaneous light perception or thermoregulation or as mediators that change during sleep (22). In humans, blood levels of stress mediators and melatonin oscillate in anti-phase with peak levels during daytime activity for stress mediators and nocturnal sleep for melatonin, respectively (430, 431). These changes induce an increase in skin temperature in the evening that in turn essentially contributes to sleep onset (44). Sleep and its associated behavioral changes further reduce nocturnal levels of stress mediators and enhance nocturnal levels of melatonin. The peripheral hormone of the HPA-axis is cortisol that regulates human keratinocyte clock functions and suppresses proliferation of these epidermal cells (49). The mediators of the SNS are the catecholamines epinephrine and norepinephrine that together with melatonin are key in thermo- and vasoregulation. Thus, the supporting effects of melatonin intake on sleep initiation is assumed to be mediated by vasodilatatory effects of this hormone (432). Cortisol, catecholamines, and melatonin also impact immune cell clocks, traffic and functions in blood and various tissues (154, 381, 390), including the skin (363). They are not only released systemically, but also produced locally in the epidermis and dermis (433, 434). In mice and rats, the peripheral hormone of the HPA-axis is corticosterone that likewise increases sharply at the transition from the rest to the activity period (435, 436). For blood and tissue levels of catecholamines in rodents, rhythms and peak times were rather inconsistent (437–439). In contrast to humans, mice show highest melatonin levels during their activity period, thus in phase with corticosterone (114). As melatonin has many effects on skin physiology and immunity (440), it therefore seems not very straightforward to study the effects of the circadian system and sleep on skin immunity in mice. A counter argument could be that many laboratory mouse strains like C57BL/6 and Balb/C seem to be genetically incapable to synthesize sufficient amounts of systemic melatonin (441) or to express melatonin receptors. C57BL/6 mice assume to lack both melatonin receptors, yet scientists bred a strain of melatonin receptor (MT)1 and MT2 knockout mice and backcrossed them into a melatonin proficient strain to study the effects of each receptor separately (442). Recently, a melatonin proficient C57BL/6 strain was also developed (443). In sum, these different mouse strains could serve to study the effects of melatonin administrations on skin immunity, but experimental design needs to be chosen with caution.

Stress mediators and melatonin may play a role in SLE (221–224), SSc (230, 231, 444), psoriasis (196, 200–202, 268, 445), Sjögren’s syndrome (234), and vitiligo (203, 209). A failure in the HPA-axis to control inflammation is discussed in autoimmune diseases (180, 248) and glucocorticoids are widely used therapeutically as immunosuppressants.

Vasodilatatory, anti-inflammatory, and melatonin-releasing effects of catecholamines are mediated by beta-adrenoceptors (389, 446). Beta-blockers therefore can have manifold unwanted effects on the circadian system, sleep, and the immune system and in this way may contribute to disease flares in psoriasis (447). Also other hormones and mediators such as growth hormone, prolactin, aldosterone, thyroid hormones, sex hormones, ghrelin, leptin, prostaglandins, serotonin, histamine, adenosine, endorphins, αMSH, neuropeptides, and vitamin D are regulated by the wake-sleep cycle (22, 35, 47, 226, 389, 390, 448), are involved in autoimmune diseases (180, 449), can impact skin physiology, and immunity (205, 209, 450) and may therefore be of relevance in the etiopathology of skin autoimmune diseases in shift workers.

As itch and pain are key, interrelated symptoms in skin autoimmune diseases, underlying mediators could be of particular interest in studying effects of shift work on dermal cells and leukocytes. Neuronal pathways of itch and pain involve A- and C-fibers in the epidermis, which are activated by histamine, neuropeptides, and cytokines, as well as forwarding of the signals *via* the dorsal root ganglion to the brain. Beta-endorphins are able to act as analgesics by binding to opiod receptors, starting a cascade of interactions, which finally results in the inhibition of pain signalling (451). Histamine is often released at the site of inflammation in the skin (452) and plasma levels of beta-endorphin are enhanced in children with atopic dermatitis (453). In healthy humans, plasma levels of beta-endorphins (but not of histamine (454)) show rhythmic regulation being highest in the morning and reduced in the night (455–457). Ligand binding to histamine receptors on leukocytes likewise changes from night to day with complex patterns in healthy and atopic individuals (458). Moreover, in healthy individuals blood levels of cortisol and beta-endorphins are coupled, meaning that cortisol follows beta-endorphin with a lag-phase of ten minutes (459). Glucocorticoids are potent antipruritic drugs, but are also known to inhibit pain pathways (460). Therefore, not only low beta-endorphin levels but also low cortisol levels could explain why symptoms of itch and pain are most pronounced at night, as it was demonstrated for itch in patients with psoriasis (211, 461).

The effect of sex hormones is expected to explain the sexual dimorphism in autoimmune diseases that predominantly affect women (462). The effect of estrogen on sleep and circadian rhythms becomes also visible in women, when they reach the menopause and suffer from sleep disorders due to a decline or imbalance of this hormone (463).

Shift workers show enhanced average cortisol levels (25) and disrupted melatonin rhythms (464). However, until yet there is no data on catecholamines, histamines, endorphins, sex hormones, or other neuroendocrine mediators in shift workers.

## 7 Countermeasures to avoid skin immune dysregulation in shift work

Until now, this review focused on potential circadian drivers of autoimmunity. However, there are also some factors that might improve and stabilize the circadian system and thereby might be able to alleviate symptoms and negative effects of shift work.

Shift work is indispensable in healthcare, public protection and transportation. Some industries use 24 h schedules due to difficulties in stopping machines and production chains. However, shift work may also serve to maximize profit. From a health perspective, night shifts should only be demanded from workers when absolutely required. To minimize health issues, employers should use sophisticated shift work schedules (i) favoring a forward rotating system instead of a backwards rotating one, (ii) allowing rest periods of at least 11 h between two shifts, days off after night shifts and free weekends, (iii) avoiding early morning starts and long night-time working hours, and (iv) limiting the number of (consecutive) evening and night shifts (465, 466). Ideally, the chronotype of the workers should be assessed (i.e., being a morning or evening person) and whenever possible, this should be taken into account when scheduling shifts (e.g., avoiding night shifts in morning persons) (467). To facilitate alignment to night shifts and re-alignment to the regular sleep-wake cycle, bright light can help to suppress melatonin secretion and sleepiness during wake periods, whereas melatonin supplementation, as well as a cool and dark bedroom with a bedding that facilitates a suitable skin temperature might help to catch up on sleep (465, 468, 469).

Melatonin acts as dark signal, which is only to some degree able to facilitate sleep. Nevertheless, the idea of melatonin supplementation is to re-gain the circadian rhythm if it was disturbed. Even though the available data on this topic is limited, there are some studies on melatonin supplementation in shift workers and also as treatment for SLE. Nabatian-Asl and colleagues were able to show that 10 mg/day melatonin supplementation for 12 weeks is reducing serum malondialdehyde, which is a marker for oxidative stress. Oxidative stress levels are known to correlate with SLE-activity (221). A recent systematic review by Carriedo-Diez and colleagues investigated melatonin supplementation in shift workers. The investigated studies used between 1 and 10 mg melatonin and recognized improvements such as reduced day-time sleepiness and increased total sleep period (470). However, the studies varied in their design and group sizes and ages. More future work will hopefully shed light on this topic, also investigating other autoimmune diseases and effects as well as side-effects of melatonin.

As food intake entrains rhythms, there are some recommendations for meal timing and composition during shifts (465, 471). Caffeine and physical activity promote wakefulness and the latter seems to protect from shift work disorder (34, 81). Pharmacological interventions in shift workers with sleeping aids such as zopiclone or wake-promoting substances such as modafinil did not lead to clear improvement of sleep or alertness, respectively (472).

The psychosocial and socioeconomic situation of the workers cannot be neglected. Apart from physical conse-quences, shift workers may also suffer from emotional and mental health issues. The socioeconomic status differs among shift workers and many report increased job stress (473) or social isolation (20). As social stress and isolation were shown to be associated with the conserved transcriptional response to adversity as an indicator of SCI (474, 475), they may mediate or influence the effects of shift work on skin autoimmune diseases. Older age, female gender, being married, or having children is increasing the risk of suffering from sleep related impairment in response to shift work (81). Medical surveillance, in particular in employees with these kind of risk factors, should also cover skin health and care. Considering working time preferences and giving employees shift schedules one month in advance can help them to plan activities with family and friends and thus to improve the work-life balance and social contacts (18, 465, 466). Further countermeasures to avoid negative health outcomes in shift workers are financial compensation, individual counselling, health education (e.g. dietary habits, physical activity, avoidance of substance abuse), and information about non-pharma-cological interventions to reduce stress (e.g., mindfulness based stress reduction) and to ease sleep (e.g. sleep hygiene, napping) (466, 476).

In case that a systemic or skin-specific autoimmune disease has already occurred, physicians might recommend to avoid night shifts, as stress and stressful events could worsen symptoms and cause further disease flares (213). If this is not feasible, wise timing of topic and/or systemic drug treatments, thus during work hours or before bedtime and not at standard clock times might be advantageous (477).

## 8 Conclusions and future directions

Available evidence indicates that shift work by disrupting the circadian system and sleep impairs skin physiology and immunity and presumably contributes to skin autoimmune diseases. Circadian and sleep aspects should be considered in basic research on immunity in particular in experiments on nocturnal animals. Moreover, other animal models such as pigs should be considered in immunologic and chronobiologic studies.

The influences of shift work and disrupted circadian clocks are manifold, ranging from fatigue and metabolic disturbances over SCI to the development of autoimmune diseases. Innate and adaptive immune cells (as well as all other cells) show rhythmic regulation and may react adversely to different stimuli such as pathogens, allergens or (auto-)antigens if the rhythm is disrupted. The signaling of the SCN downwards to peripheral organs and cells is mediated by hormones like melatonin and cortisol. These hormones should be monitored in shift workers, to delineate their role in the development of autoimmune diseases. The skin as the largest human organ is in contact with the outer world and therefore an important barrier, which seems to be weakened by circadian disruption, sleep loss, or shift work. In our society, shift work is unavoidable, however, this review offers several opportunities to improve the health of shift workers.

## Author contributions

All authors contributed in writing and revising the manuscript. TL and JH developed the concept, structure and both tables, SS prepared the figures and wrote paragraphs about animal studies and B cells, HG provided her clinical insight and knowledge especially regarding psoriasis, arthritis and inflammatory bowel disease and wrote these sections. All authors contributed to the article and approved the submitted version.
